# Supplementary material for: G-quadruplex structures regulate long-range transcriptional reprogramming to promote drug resistance in ovarian cancer cells
Source: Genome Biol. 2025 Jul 12;26:183. doi: 10.1186/s13059-025-03654-y (PMC12255116; doi:10.1186/s13059-025-03654-y)
Supplement: Supplementary file 1 — Additional file 1: Figs. S1–S11. [file 13059_2025_3654_MOESM1_ESM.pdf]

Additional file 1

G-quadruplex structures regulate long-range transcriptional reprogramming to promote drug resistance in ovarian cancer

Jenna Robinson, Gem Flint, Ian Garner, Silvia Galli, Thomas E. Maher, Marina K. Kuimova, Ramon Vilar, Iain A. McNeish, Robert Brown, Hector Keun, Marco Di Antonio

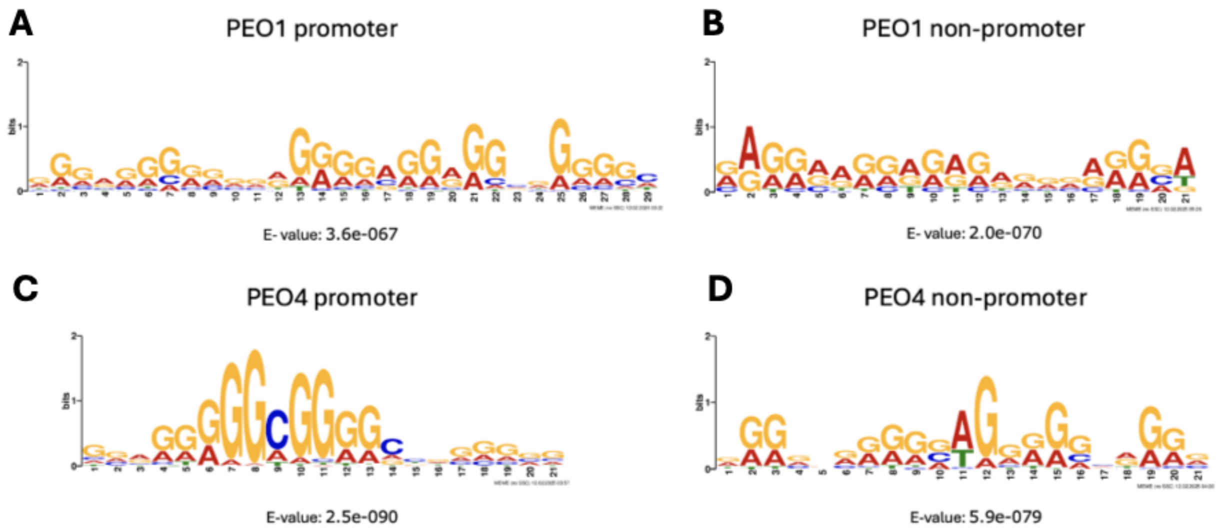

Figure S1 – Top motifs enriched in BG4 ChIP-seq peaks in A) PEO1^{BRCA-} promoters, B) PEO1^{BRCA-} non-promoter sites, C) PEO4 promoters and D) PEO4 non-promoter sites PEO4. Motifs discovered with MEME.

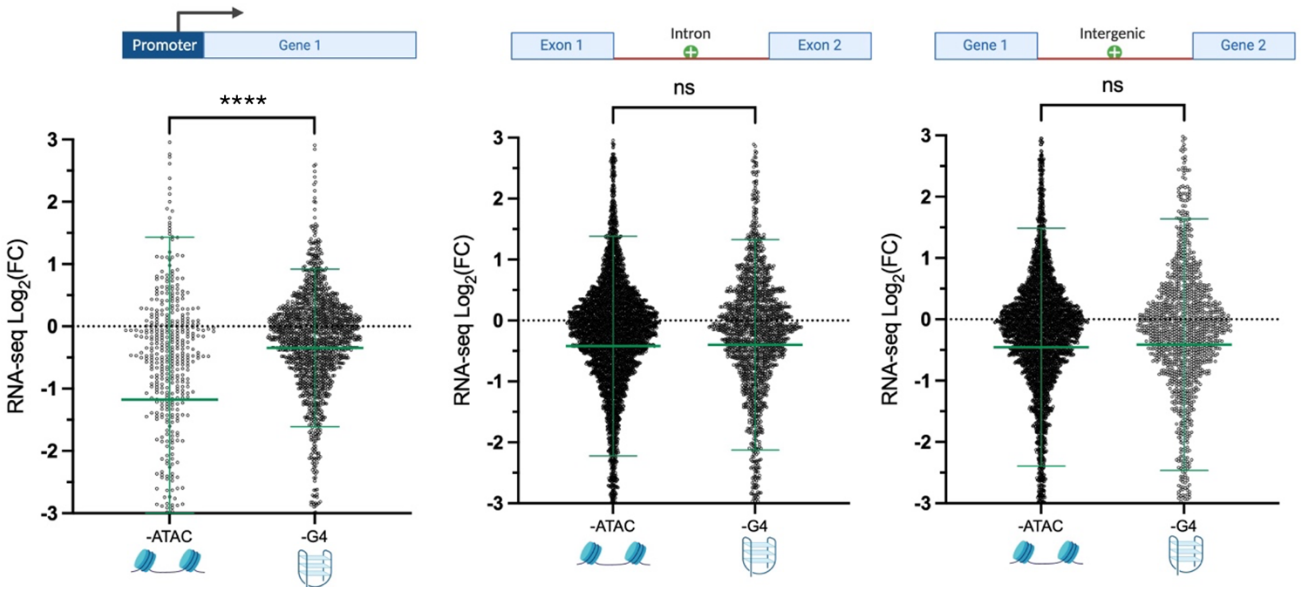

Figure S2 – Change in expression of genes that lose an ATAC or G4 ChIP-seq peak in PEO4 relative to $PEO1^{BRCA-}$, at promoters, intergenic and intronic regions. Only ATAC-peaks that overlap OQS (identified by G4-seq) were examined. Statistical significance assessed by Mann-Whitney U-test. FC= fold-change; ns = non-significant; **** = p<0.0001.

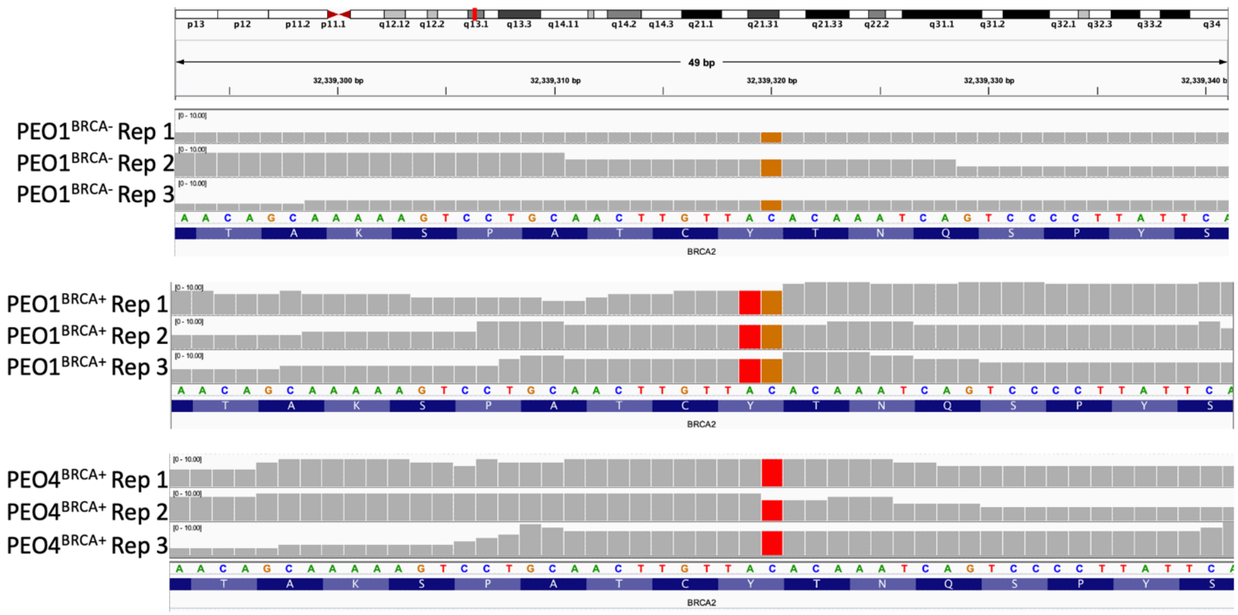

Figure S3 – IGV profiles of RNA-seq data in $PEO1^{BRCA-}$, $PEO1^{BRCA+}$ and $PEO4^{BRCA+}$ cells, showing exon 11 of BRCA2. $PEO1^{BRCA-}$ harbor a single C>G point mutation which results in the premature introduction of a stop codon, $PEO1^{BRCA+}$ contain an additional A>T mutation restoring BRCA2 expression and $PEO4^{BRCA+}$ a neutral C>T alteration. Data from three biological replicates is shown.

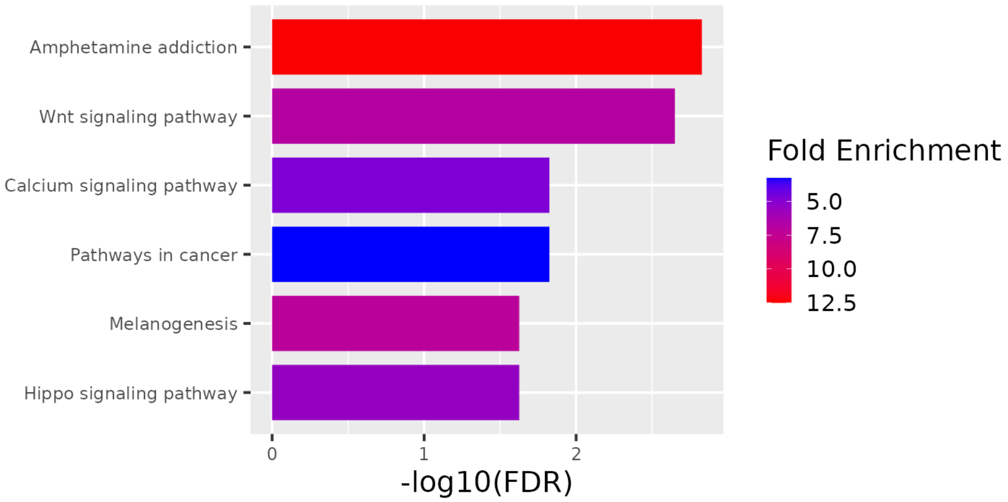

Figure S4– Enriched KEGG pathways of upregulated genes associated with new intergenic/intronic G4s in PEO4 (relative to PEO1^{BRCA+}). Enrichment analysis performed with ShinyGO.

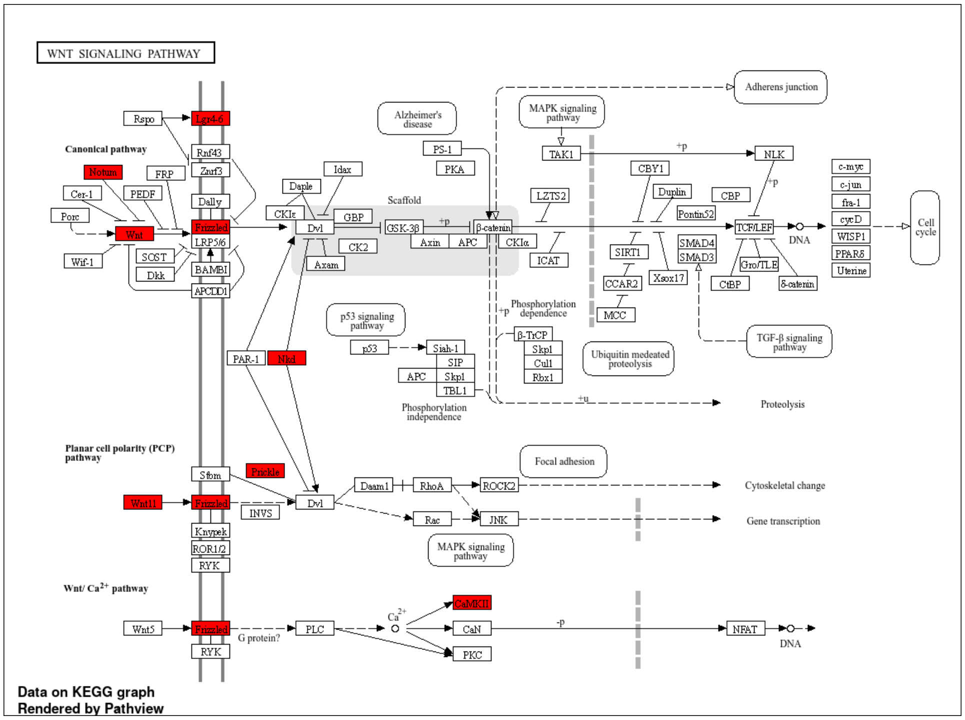

Figure S5 – Proteins involved in WNT signaling pathway. Proteins in red are significantly upregulated in PEO4 relative to PEO1^{BRCA+} and are proximal to PEO4-specific intergenic/intronic G4 CUT&Tag peaks.

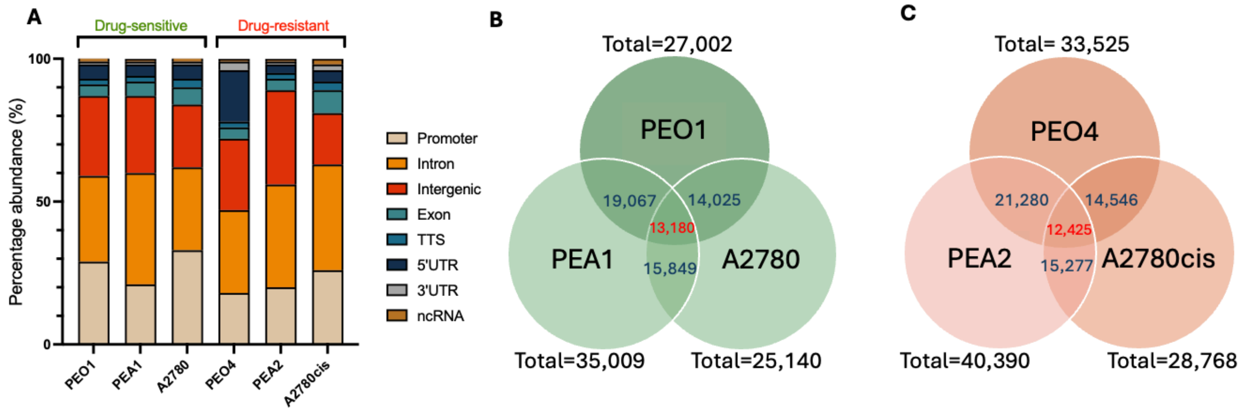

Figure S6 - A) Genomic distribution of accessible G4 sequences in drug-sensitive (PEO1, PEA1, A2780) and drug-resistant (PEO4, PEA2, A2780cis) cells. B) Overlap between accessible G4 sequences in PEO1, PEA1 and A2780. C) Overlap between accessible G4 sequences in PEO4, PEA2 and A2780cis.

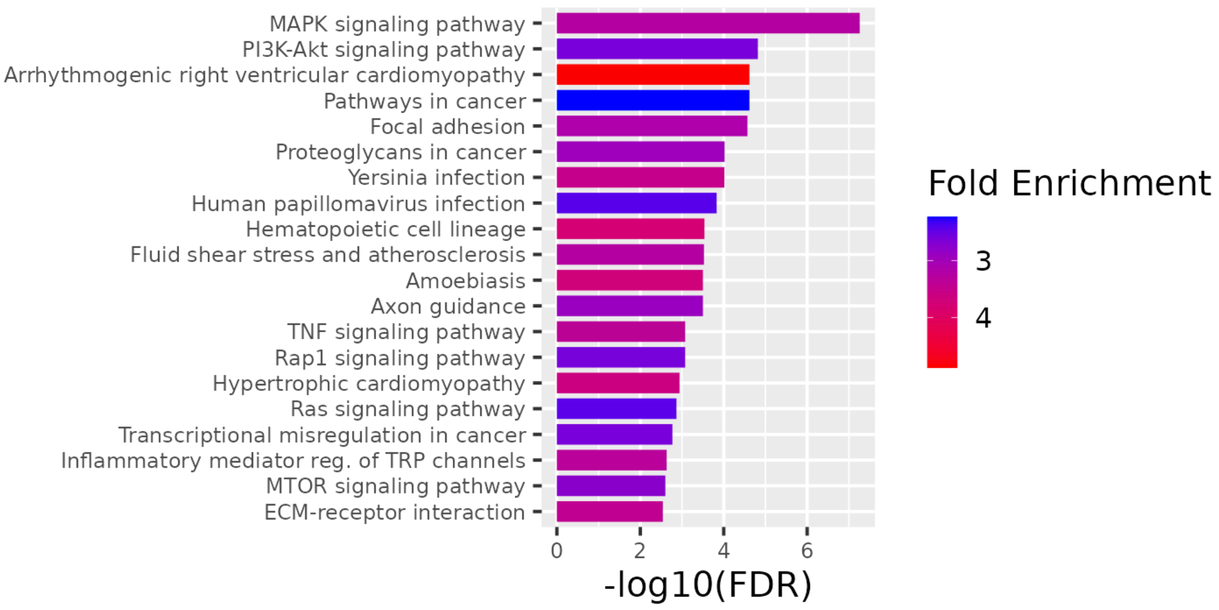

Figure S7 – Enriched KEGG pathways for significantly upregulated genes in PEA2 (relative to PEA1), associated with new ATAC peaks that contain OQS (as defined by G4-seq). Enrichment analysis performed with ShinyGO.

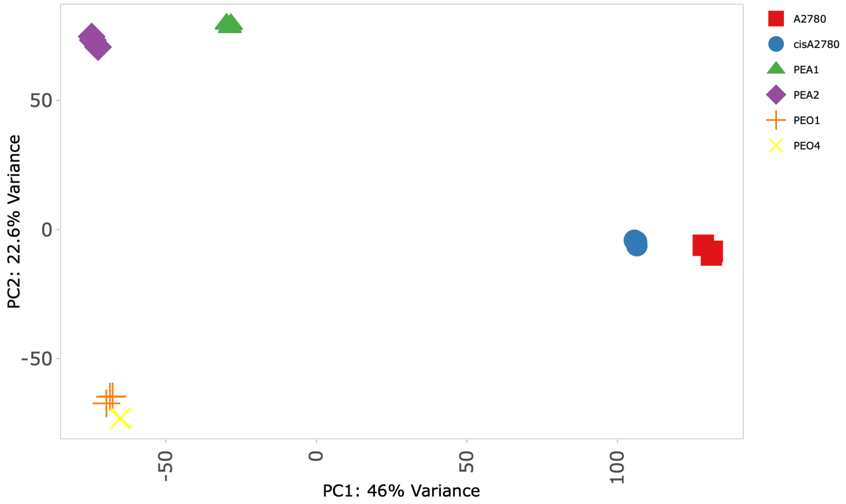

Figure S8 – Principal component analysis of RNA-seq data in PEO1, PEO4, PEA1, PEA2, A2780 and cisA2780. Analysis performed in iDEP.

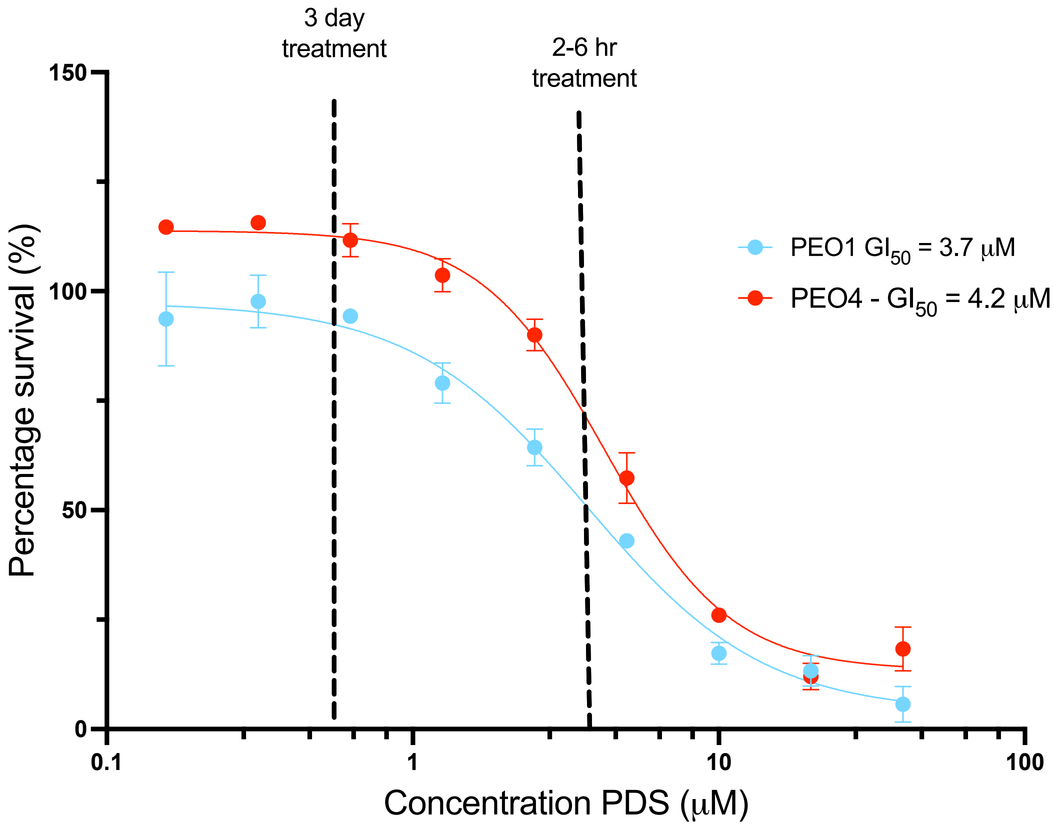

Figure S9– MTS viability assay of PEO1^{BRCA+} (green) and PEO4 (red) cells in response to increasing concentrations of PDS, incubated for 72 hours. Dotted lines show concentrations used for 3-day synergy experiments with cisplatin (0.5 μM) and for 2/6 hour PDS treatment followed by RNA-sequencing (5 μM). Error bars are standard deviations for experiments performed in triplicate.

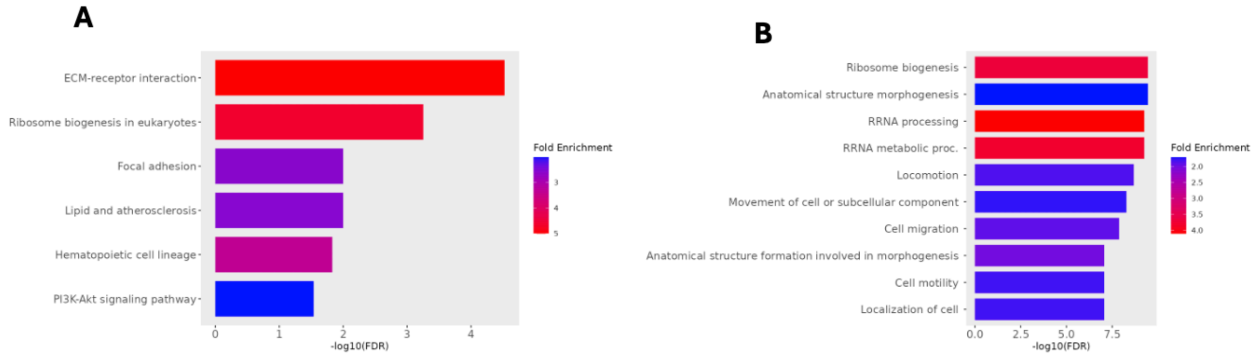

Figure S10 – A) Significantly enriched KEGG pathways and B) GO biological processes of genes significantly upregulated in PEO4 after PDS treatment (6 hours).

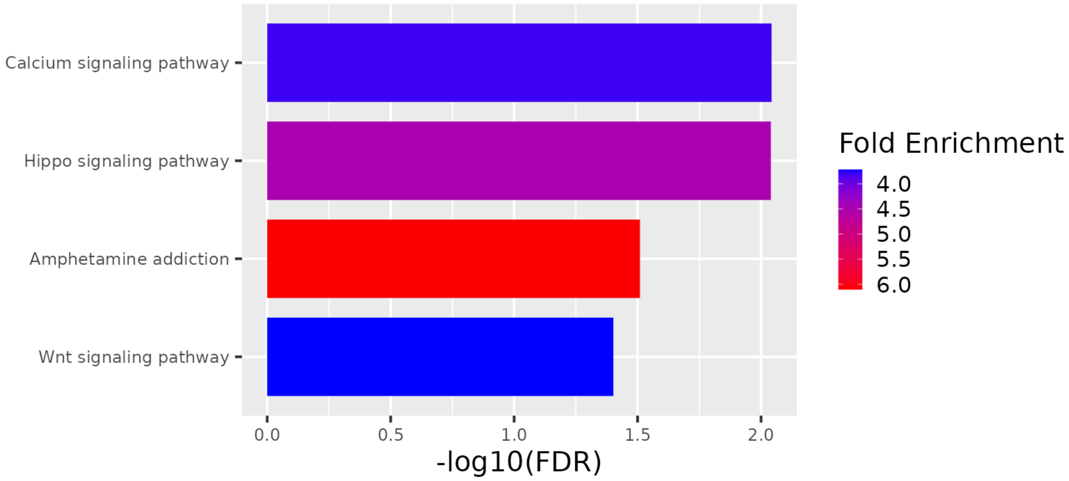

Figure S11 – KEGG pathways enriched in genes containing intergenic/intronic G4s (detected by BG4 CUT&Tag) in PEO4 that were significantly down-regulated after 6 hours PDS treatment (5 μM). Enrichment analysis performed with ShinyGO.
