# Supplementary material for: G-quadruplex structures regulate long-range transcriptional reprogramming to promote drug resistance in ovarian cancer cells
Source: Genome Biol. 2025 Jul 12;26:183. doi: 10.1186/s13059-025-03654-y (PMC12255116; doi:10.1186/s13059-025-03654-y)
Supplement: Supplementary file 2 — Additional file 2: Tables S1–S7. [file 13059_2025_3654_MOESM2_ESM.pdf]

Additional file 2

G-quadruplex structures regulate long-range transcriptional reprogramming to promote drug resistance in ovarian cancer

Jenna Robinson, Gem Flint, Ian Garner, Silvia Galli, Thomas E. Maher, Marina K. Kuimova, Ramon Vilar, Iain A. McNeish, Robert Brown, Hector Keun, Marco Di Antonio

Table S1 - Enriched hallmarks associated with upregulated genes proximal to new intergenic/intronic G4s in PEO4 relative to PEO1^{BRCA+}. Enrichment analysis performed with GSEA.

**Pathway**	**FDR**
Estrogen response late	1.0 e^{-8}
Estrogen response early	9.5 e^{-7}
KRAS signaling down	7.9 e^{-5}
TNGA signaling via NFKB	5.6 e^{-4}
Myogenesis	3.6 e^{-3}
UV response UP	7.0 e^{-3}
WNT-Beta catenin signaling	7.0 e^{-3}
Epithelial mesenchymal transition	1.6 e^{-2}

Table S2 – Top 3 publications containing genesets most similar to upregulated genes associated with new intergenic/intronic G4s in PEO4 (relative to PEO1^{BRCA+}), identified with NDEx iquery, pathway figures.

**Publication**
DNA methylation and Transcriptome Changes Associated with Cisplatin Resistance in Ovarian Cancer, Lund et al, 2017
Methylome-wide Sequencing Detects DNA Hypermethylation Distinguishing Indolent from Aggressive Prostate Cancer, Bhasin et al., 2015
The clinical applications of the cancer genome atlas project for bladder cancer, Creighton et al., 2018

Table S3 – Top 3 publications containing genesets most similar to upregulated genes associated with new promoter G4s in PEO4 (relative to PEO1^{BRCA+}), identified with NDEx iquery, pathway figures.

Publication
Identification of Targets of CUG-BP, Elav-Like Family Member 1 (CELF1) Regulation in Embryonic Heart Muscle, Blech-Hermoni et al., 2016
Differential Effects of Human SP-A1 and SP-A2 on the BAL Proteome and Signaling Pathways in Response to Klebsiella pneumoniae and Ozone Exposure, Wang et al., 2019
Identification of Important Effector Proteins in the FOXJ1 Transcriptional Network Associated With Ciliogenesis and Ciliary Function, Mukherjee et al., 2019

Table S4 – Enriched hallmarks for top 500 genes significantly upregulated in PEA2 (relative to PEA1) associated with new ATAC peaks that contain OQS (as defined by G4-seq). Enrichment analysis performed with GSEA.

Pathway	FDR
KRAS signaling up	3.65 e-20
Inflammatory response	8.29 e-14
Estrogen response early	4.50 e-13
TNFA signaling via NFKB	4.50 e-13
Epithelial to mesenchymal transition	2.74 e-10
Estrogen response late	2.74 e-10
Apoptosis	6.64 e-10
Apical junction	1.78 e-9
Complement	1.28 e-8
Allograft rejection	6.14 e-7

Table S5 – Primer sequences used for BG4 ChIP qPCR

Primer region	Sequence (5'-3')
MAZ forward (G4 region)	ACT CAG CGC AGG ATT GTA AAT A
MAZ reverse (G4 region)	CCT CAT GCT TCG GCT TCC
RPA3 forward (G4 region)	CGG AAG TTG ACA GAT ACA GGG
RPA3 reverse (G4 region)	GAT CGC AGA AAG GTA GTC TCA G
KIF14 forward (G4 region)	CGG TAG CCG TCT CTG AAT G
KIF14 reverse (G4 region)	CTT TAG CAG AAC CCG AGG AG
SPRED2 forward (G4 region)	AAC AGG AGG AGG AAG TAG GG
SPRED2 reverse (G4 region)	TTT CGG TCG CAA GTA GGA AG
TMCC1 forward (non-G4 region)	GTG GTA CAC TGC CTA CAG TAT T
TMCC1 reverse (non-G4 region)	GTA TAA CGC CTG GGC TAT GT
IL36G forward (non-G4 region)	GCC CAC CTC TTT ACT TCC TTA
IL36G reverse (non-G4 region)	AAC ACT CTT TCA GCT CCA TCC

Table S6– Primer sequences used for BG4 CUT&Tag of PEO1^{BRCA+} and PEO4

**Primer name**	**Sequence**
universal i5	AATGATACGGCGACCACCGAGATCTACACTAGATCGCTCGTCGGCAGCGTCAGATGTGTAT
i7_1	CAAGCAGAAGACGGCATACGAGATTCGCCTTAGTCTCGTGGGCTCGGAGATGTG
i7_2	CAAGCAGAAGACGGCATACGAGATCTAGTACGGTCTCGTGGGCTCGGAGATGTG
i7_3	CAAGCAGAAGACGGCATACGAGATTTCTGCCTGTCTCGTGGGCTCGGAGATGTG
i7_4	CAAGCAGAAGACGGCATACGAGATGCTCAGGAGTCTCGTGGGCTCGGAGATGTG
i7_5	CAAGCAGAAGACGGCATACGAGATAGGAGTCCGTCTCGTGGGCTCGGAGATGTG
i7_6	CAAGCAGAAGACGGCATACGAGATCATGCCTAGTCTCGTGGGCTCGGAGATGTG
i7_7	CAAGCAGAAGACGGCATACGAGATGTAGAGAGGTCTCGTGGGCTCGGAGATGTG
i7_8	CAAGCAGAAGACGGCATACGAGATCCTCTCTGGTCTCGTGGGCTCGGAGATGTG
i7_9	CAAGCAGAAGACGGCATACGAGATAGCGTAGCGTCTCGTGGGCTCGGAGATGTG
i7_10	CAAGCAGAAGACGGCATACGAGATCAGCCTCGGTCTCGTGGGCTCGGAGATGTG
i7_11	CAAGCAGAAGACGGCATACGAGATTGCCTCTTGTCTCGTGGGCTCGGAGATGTG
i7_12	CAAGCAGAAGACGGCATACGAGATTCCTCTACGTCTCGTGGGCTCGGAGATGTG

Table S7– Primer sequences used for ATAC-seq of PEO1^{BRCA-} and PEO4

**Primer name**	**Primer sequence (5'-3')**
Ad1_noMX (Primer 1)	AATGATACGGCGACCACCGAGATCTACACTCGTCGGCAGCGTCAGATGTG
Ad2.1_TAAGGCGA (Primer 2)	CAAGCAGAAGACGGCATACGAGATTCGCCTTAGTCTCGTGGGCTCGGAGATGT
Ad2.2_CGTACTAG (Primer 2)	CAAGCAGAAGACGGCATACGAGATCTAGTACGGTCTCGTGGGCTCGGAGATGT
Ad2.3_AGGCAGAA (Primer 2)	CAAGCAGAAGACGGCATACGAGATTTCTGCCTGTCTCGTGGGCTCGGAGATGT
Ad2.4_TCCTGAGC (Primer 2)	CAAGCAGAAGACGGCATACGAGATGCTCAGGAGTCTCGTGGGCTCGGAGATGT
Ad2.6_TAGGCATG (Primer 2)	CAAGCAGAAGACGGCATACGAGATCATGCCTAGTCTCGTGGGCTCGGAGATGT
Ad2.9_GCTACGCT (Primer 2)	CAAGCAGAAGACGGCATACGAGATAGCGTAGCGTCTCGTGGGCTCGGAGATGT
Ad2.11_AAGAGGCA (Primer 2)	CAAGCAGAAGACGGCATACGAGATTGCCTCTTGTCTCGTGGGCTCGGAGATGT
Ad2.12_GTAGAGGA (Primer 2)	CAAGCAGAAGACGGCATACGAGATTCCTCTACGTCTCGTGGGCTCGGAGATGT
